# Assessment of anammox, microalgae and white-rot fungi-based processes for the treatment of textile wastewater

**DOI:** 10.1371/journal.pone.0247452

**Published:** 2021-03-02

**Authors:** Micol Bellucci, Francesca Marazzi, Alida Musatti, Riccardo Fornaroli, Andrea Turolla, Simone Visigalli, Martina Bargna, Giovanni Bergna, Roberto Canziani, Valeria Mezzanotte, Manuela Rollini, Elena Ficara

**Affiliations:** 1 Politecnico di Milano, DICA, Milan, Italy; 2 Università degli Studi di Milano-Bicocca, DISAT, Milan, Italy; 3 Università degli Studi di Milano, DeFENS, Milan, Italy; 4 Lariana Depur SPA, Fino Mornasco, CO, Italy; Luleå University of Technology, SWEDEN

## Abstract

The treatability of seven wastewater samples generated by a textile digital printing industry was evaluated by employing 1) anammox-based processes for nitrogen removal 2) microalgae (*Chlorella vulgaris)* for nutrient uptake and biomass production 3) white-rot fungi (*Pleurotus ostreatus* and *Phanerochaete chrysosporium)* for decolorization and laccase activity. The biodegradative potential of each type of organism was determined in batch tests and correlated with the main characteristics of the textile wastewaters through statistical analyses. The maximum specific anammox activity ranged between 0.1 and 0.2 g N g VSS^-1^ d^-1^ depending on the sample of wastewater; the photosynthetic efficiency of the microalgae decreased up to 50% during the first 24 hours of contact with the textile wastewaters, but it improved from then on; *Pleurotus ostreatus* synthetized laccases and removed between 20–62% of the colour after 14 days, while the enzymatic activity of *Phanerochaete chrysosporium* was inhibited. Overall, the findings suggest that all microbes have great potential for the treatment and valorisation of textile wastewater after tailored adaptation phases. Yet, the depurative efficiency can be probably enhanced by combining the different processes in sequence.

## Introduction

The textile sector has a severe environmental impact, consuming 79 billion cubic metres of water and emitting more than 1.7 million tons of CO_2_ in the EU [1]. The amount of water consumed, and consequently the polluted water released, is strictly dependent on the requirement of the different stages of the production chain, type of fabric produced and machines utilized [2]. The dyeing and printing steps are of major concern as they generate wastewaters rich in organic pollutants (as chemical oxygen demand, COD), nutrients (nitrogen and phosphorus), heavy metals, dyestuffs, catalytic chemicals, and cleaning solvents, which are extremely toxic for the environment and human beings [3]. The implementation of the digital textile printing (DTP) technology in several European companies has substantially decreased the environmental impact of the textile sector, whilst enhancing the quality level in a wide range of fabrics. Compared to conventional processes, DTP consumes less water and energy, emits less CO_2_, and requires less chemicals [4–6]. Nevertheless, the ink-jet printing procedure includes a pre-impregnation of the fabrics in urea, which increases the load of organic nitrogen (up to 600 mg N L^-1^) in the discharged wastewater.

Several physicochemical, biological, and combined treatment processes have been extensively used for the removal of pollutants in textile wastewaters (TW) [7,8]. However, the best available technologies, including adsorption on activated carbon, addition of iron salts to the activated sludge, and ozonation, are expensive and/or energy intensive [9]. On the other hand, the conventional process for biological nitrogen removal of such N-rich wastewater needs intensive aeration and organic substrates, with the drawback of further consumption of energy, production of large amounts of sludge and massive emission of green-house gases. In this light, the development of novel cost-effective bioremediation technology is essential to improve the environmental sustainability of the textile industry.

Recently, the completely autotrophic nitrogen removal (ANR) process emerged as an effective alternative for the treatment of textile wastewater from DTP companies [10]. ANR relies on the combined activity of the ammonium oxidizing bacteria (AOB), which convert part of the ammonia into nitrite, with the anaerobic ammonium oxidation (anammox) bacteria (AMX) that transform the remaining ammonia and nitrite into N_2_ and water. The process has no demand of organic carbon and lower demand of oxygen compared to conventional biological nutrient removal processes, and it is thus a more cost effective and environmentally friendly alternative [11,12]. Surveys carried out in 20 years of application of ANR for the treatment of several types of wastewaters show that the main drawback of this process is that the AMX are extremely sensitive to operational and environmental conditions [13–15]. Therefore, tailored studies focusing on the treatability of TW by AMX are needed to assess its applicability to the textile sector [10].

Microalgae and cyanobacteria have received much attention for the treatment and valorisation of domestic and industrial wastewater, including textile wastewater [16–19]. Microalgae can remediate TWs by uptaking nutrients (nitrogen and phosphorus) and heavy metals, but also by removing colour through bioadsorption, biodegradation and bioconversion [20–22]. In addition, the microalgal biomass produced during the process can be exploited as a source of polysaccharides, lipids, pigments, proteins, bioactive compounds [23]. Colour removal efficiency by microalgae and cyanobacteria can reach up to 95% according to the species and cellular state of the microalgae and to the molecular structure of the dye [24]. *Chlorella vulgaris*, *Chlorella pyrenoidosa*, *Scenedesmus quadricauda*, *Oscillatoria curviceps* have been reported to efficiently biodegrade and decolorize several types of azo dyes, including supranol red, acid black, Orange II, Remazol Brilliant Blue [19,25,26]. Colour and ammonia removal by *C*. *vulgaris* in a high rate algae pond (HRAP) fed on undiluted TW reached up to 47% and 45% respectively after 12 days of cultivation in batch [19]. Nonetheless, few experiences are reported about long term cultivation of microalgae in raw TW and mostly concern the association of microalgae with other physicochemical or microbial processes [27–29].

The application of white-rot fungi has great potential for cost-effective decolorization of textile wastewaters. These fungi synthetize several intra and extracellular ligninolytic enzymes including laccase (LC), Mn-peroxidase (MnP) and lignin-peroxidase (LiP), which allow the degradation of several complex organics, such as azo-dyes [30]. In optimal growing conditions, immobilized *Phanerochaete chrysosporium* and *Pleurotus ostreatus* have been reported to efficiently decolorize (80–100% removal) solutions with a broad range of acidic and reactive dyes (25–200 ppm) such as Acid Red 88, Reactive Black 5 and Reactive Orange 16 and Orange II [31–33]. Nevertheless, the physicochemical conditions, especially pH, of the TW might impair the enzymatic pathways. This is probably the reason why studies dealing with fungal treatment of real TWs (not model solutions) are still scarce [34].

As stated above, anammox bacteria, microalgae and fungi have great bioremediation potential, though their activity depends on the characteristics of the TWs. Even effluents of the same industry face changes in type and concentration of recalcitrant and toxic compounds according to seasons, fashion markets, type of fabrics and finished textile products. Therefore, to consider these microbes for the development of a solid and reliable bioremediation process, it is essential to assess how the variability of the TW composition impacts their growth and activity. The goal of the present study is to assess the potential application of processes based on anammox bacteria, microalgae, and fungi for the treatment of a real TW, derived by a textile digital printing company. To accomplish such scope: 1) the physicochemical composition of textile wastewater samples has been monitored for two months to assess its variability; 2) the performance and efficiency of the biological processes carried out by the three groups were evaluated and compared; 3) the physicochemical characteristics of the tested wastewater samples were correlated with the activities of each type of organism through statistical tools.

## Materials and methods

### Wastewater origin and sampling

Wastewater samples were collected in a textile company, which is placed in Northern Italy (Stamperia di Cassina Rizzardi Spa, Como). The company has a printing capacity of 9,000,000 m year^-1^, operates 28 ink-jet printers and discharges more than 430,000 m^3^ year^-1^ of wastewater. All the effluents of the manufacturing chain are collected in an equalization tank (1200 m^3^) where the wastewater is stored and eventually discharged into the sewage network (1000 m^3^ day^-1^). An automatic sampler takes 200 mL every 20 m^3^ discharged to allow quality controls of the effluent. During the wastewater sampling campaign, daily aliquots were collected within a week and mixed to obtain a single, homogeneous and representative weekly sample (1.4 L, n = 7, TW1-TW7). Part of the composite sample (100 mL) was immediately analysed for its physicochemical properties while the rest was stored at -20°C until the execution of the parallel bioremediation essays with the three organisms.

### Analytical methods

Total and volatile suspended solids (TSS and VSS) in the wastewater samples were determined in duplicate according to Standard Methods [35]. Conductivity and pH were measured by a portable probe (XS PC 510 Eutech Instruments, Stevensville, MI, USA). Concentrations of NH_4_^+^-N, NO_3_^—^N, NO_2_^—^N and total nitrogen (TN-N), orthophosphate phosphorus (PO_4_^-3^-P) and soluble COD (sCOD) were determined on filtered (0.45 μm) TW samples by spectrophotometric test kits (DR6000TM UV VIS Spectrophotometer, Hach Lange LT200 Dry thermostat, Germany). COD of not filtered samples were also determined. The concentration of free NH_3_-N was computed from the NH_4_^+^-N according to Anthonisen et al. [36].

Colour characterization was based on the optical density spectrum of filtered samples in a 1 cm cuvette by a UV-spectrophotometer (Hach-Lange, DR6000TM UV VIS Spectrophotometer), while the total colour was estimated by summing the optical density at 445 nm, 540 nm, 660 nm as described by Pala et al. [37]. Turbidity was measured by the optical density at 780 nm wavelength in a 5 cm cuvette. Cu, Cr, Cd, Pb and Ni concentrations in the TW were determined by Graphite Furnace Atomic Absorption Spectrometry (GFAAS; SIMAA 6000, PerkinElmer) (IRSA-CNR: 3250, 3150,3220), while Zn, K, Mg, Ca, Si, Fe, Na, Mn, Mo and Al by Inductively Coupled Plasma-Optical Emission Spectroscope (ICP-OES; Optima 7000 DV PerkinElmer, Software control WinLab) (IRSA-CNR 4020 and 3030).

### Bioremediation tests

#### Anammox bioassay

Short term batch inhibition tests lasting about two/three days were performed to quantify the potential reduction of the AMX activity upon exposure to the seven TW samples. The maximum specific anammox activity (SAA^max^) of granular biomass, which was provided by PAQUES (The Netherlands) was determined by a manometric procedure [38]. For this purpose, OxiTopControl equipment (WTW, Germany) consisting in air-tight closed bottles with a volume of approximately 335 mL, equipped with a pressure detector for data acquisition and storage were implemented. Each bottle includes two air-tight rubber septa for the injection of substrates, regulation of pressure, and sampling of the medium (S1 Fig). All tests were carried out in duplicate by inoculating 5.12 g (wet weight, 0.3 g volatile solids) of anammox granules and 220 mL of wastewater. Control tests, in which the wastewater was replaced by an optimal substratum for the growth of anammox bacteria (SM) [39] were also conducted. Once set up, the bottles were flushed with a mixture of N_2_ and CO_2_ gas to remove the oxygen, then incubated at 30°C and constantly stirred (100 rpm).

The biological process taking place inside the bottle was estimated by monitoring the overpressure increase due to the release of N_2_ gas after injections (spikes) of a solution containing 50 mg L^-1^ of nitrite, which is the limiting substrate for the activity of the anammox bacteria in the TW samples. One spike (spike 1) was performed after one hour of incubation, while a second one (spike 2) was performed after 2 days from inoculation.

The pressure data collected after each injection were then converted into moles of nitrogen produced using the ideal gas equation as follows:

$$n_{N_{2}}(t)=\frac{P(t){\times V}_{HS}}{R\times T}$$

Where *V_HS_* is the volume of the gaseous phase (L) and *R* is the ideal gas coefficient (atm L mol^-1^ K^-1^), *T* is the temperature (K) and *P(t)* is the pressure (atm) at time *t* (h).

The $n_{N_{2}}(t)$ trend shows an initial positive slope (m_1_) representing the nitrogen gas production by AMX (and potentially denitrifying bacteria, carrying out the conversion of nitrite and nitrate into N_2_). Once nitrite is consumed, the curve flattens, evidencing a reduced slope (m_2_) which is due to the nitrogen gas production rate by the sole denitrifying bacteria on nitrate. Therefore, the nitrogen gas produced by the sole AMX is given by:

$${SSA}^{max}[\frac{g\;N2-N}{g\;VS\;d}]=\frac{\left(m_{1}-m_{2})[\frac{mol}{h}]\times 28[\frac{g\;N2}{mol}\right]}{M_{VS}[g\;VS]}\times 24[\frac{h}{d}]$$

Where M_VS_ is the mass of volatile solids is the granular inoculum.

#### Microalgal bioassay

This assay was carried out to evaluate the growth of microalgae on the TW samples. A pure culture of *Chlorella vulgaris* (211-11j from University of Goettingen, previously grown on Basal Medium at the laboratory of Istituto Lazzaro Spallanzani, Italy) was used as inoculum for cultivation batch tests (duplicate). The microalgal culture was mildly centrifuged to remove the supernatant, while the resulting algal biomass pellet was resuspended in 0.2 L of each TW sample and in 0.2 L of Modified Bold’s Basal Medium (MBBM) [40] (with 200 mg N L^-1^ of ammonium as the sole source of nitrogen (control test) to obtain an initial absorbance of 0.13 ± 0.03 at 680 nm wavelength. No pH adjustment was done because *Chlorella* sp. can grow in a wide range of pH (4–10) [41]. All tests were conducted for 15 days at room temperature (22 ± 2°C) using 0.5 L baffled flasks, continuously mixed by an orbital shaker (IKA, KS 501, Staufen Germany) rotating at 120 rpm. Light was provided by 4 Fluora lamps (36 W) at 200 μmol m^–2^ s^–1^ PAR, with light/dark cycles:12 h/12 h.

Total suspended solids (TSS) and volatile suspended solids (VSS), pH and the concentration of N compounds in the microalgal suspensions were measured weekly with the same procedures described above for the TW samples.

Photosynthetic activity of the cultures was assessed by phyto-PAM II (Heinz Walz GmbH, Effeltrich, Germany), which measures the photosynthetic efficiency (Fv/Fm) that represents the quantum efficiency of Photo-system II [42]. This method, which is usually applied to investigate the stress factors (e.g. photoinhibition, light limitation, temperature stress) affecting the photosynthetic rate of algal cultures [43], was used here to assess and compare the physiological state of the microalgae grown on textile wastewater and control medium. This parameter was determined according to Marazzi et al. [17]. Microalgal suspensions were diluted in order to get an absorbance of 0.1 at 680 nm and adapted to dark conditions for 20 min prior to measurement. The photosynthetic efficiency was evaluated at the beginning of the tests, after 1, 48 and 72 h, 1 and 2 weeks and compared with the control as follows:

$$Fv/Fm\%=\frac{\left(Fv/Fm\;C-Fv/Fm\;TW\right)}{Fv/Fm\;C}*100$$

Where C is the control and TW is the textile wastewater sample.

Recovery time was defined as the time needed to the microalgae to reach a photosynthetic activity similar to control: Fv/Fm % > 75. The photosynthetic activity at the different times was linearly interpolated and the recovery time was calculated as the smaller time with a Fv/Fm% higher than 75.

The average efficiency of algal production was calculated as follows:

$$ɳVSS=\frac{{VSS}_{T0}-{VSS}_{Te}}{{VSS}_{T0}}*100$$

Where VSS_T0_ and VSS_Te_ are the VSS concentration measured at the beginning and at the end of the batch test. Similar equations were used to calculate the average efficiency for the removal ammonium (ɳNH_4_^+^-N), and total nitrogen (ɳTN-N), and for the production of total suspended solids (ɳTSS) and absorbance (ɳABS).

The cellular state and size were also visualized by an optical microscope (B 350, Optika, Italy) under the 40x objective. Images were captured by a camera installed on the microscope and then processed by ImageJ (https://imagej.nih.gov/ij/).

#### Fungi bioassay

The scope of this bioassay was to examine the fungi decolorization potential on the seven TW samples. *Pleurotus ostreatus* ATCC 96997 (ATCC: American Type Culture Collection, Manassa, USA) and *Phanaerochaete chrysosporium* DSM 9620 (DSMZ: Deutsche Sammlung von Mikroorganismen und Zellkulturen GmbH, Braunschweig, Germany) were maintained on 5 cm plates containing PDA (Potato Dextrose Agar) medium (Formedium, England) as reported by Musatti et al. [44] until further use. For the bioassay, a quarter (around 5 cm^2^) of a pre-grown solid culture (not older than 1 months), taken off with a sterile scalpel, was first inoculated in 500 mL Erlenmeyer flasks containing 100 mL of liquid MEB (Malt Extract Broth) medium having the following composition (g L^-1^): glucose (Duchefa, Haarlem, the Netherlands) 20, soybean peptone (Costantino, Favria, Italy) 1, malt extract (Costantino) 20, distilled water to 1 L, pH 5.8. Flasks were incubated at 25°C on an alternative shaker (40 spm, 4 cm run) in the dark for 5 days to obtain a visible growth (presence of pellets).

Pre-cultures grown on glucose were then employed as inoculum (10% v/v) in 500 mL Erlenmeyer flask containing 100 mL of each of the TW effluents (pH was reduced to 7 with 1 M HCl), added with 1 g L^-1^ yeast extract (Costantino) and 20 g L^-1^ glucose to support fungal growth and enzymatic synthesis. Cultures were incubated at 25°C on an alternative shaker (40 spm, 4 cm run) in the dark up to 14 days.

Culture pH, effluent colour removal (%) and laccase activity (IU L^-1^) were monitored after 5–10 and 14 days [45]. At 14 days, mycelia were separated from the culture supernatants by filtration and dried at 105°C for 24 h to determine the total solid content (g L^-1^). Effluent colour removal was determined spectrophotometrically by summing the optical densities at 445 nm, 540 nm and 660 nm and calculating the colour removal efficiency with respect to *t*_*0*_ [37]. Laccase was determined by oxidation of 2,20 -azino-bis-(3- ethylbenzothiazoline)-6-sulfonic acid diammonium salt (ABTS, Fluka, Sigma Life Science, Steinheim, Germany) (Ɛ420: 36000 mol^-1^cm^-1^). Qualitative laccase activity was determined as reported by More et al. [46] with some modifications. Briefly, 200 μL of each sample were poured in wells made on (5x5 cm) plates containing 15 mL of 0.1 M sodium acetate buffer pH 4.5 added with 0.5 mM ABTS solidified with agarose (0.05 g L^-1^). Plates were incubated at 37°C for 30 min and the development of an intense bluish-green colour around the wells was considered as a positive test for laccase activity.

Samples showing positive response in qualitative trials were then subjected to laccase uantification. Trials were performed in 96-well plates, applying the procedure of Li et al. [45] with some modifications. Each well was filled with the following solutions in the ratio 1:1:1 (in volume): i) sample properly diluted, iii) 0.1 M sodium acetate buffer pH 5.0, iii) 0.5 mM ABTS in the same acetate buffer. Plates were incubated at 37°C for 10 min in a microplate spectrophotometer (MicroWave RS2, Biotek, USA) and Gene5 software (Biotek, USA) and the increase of absorbance monitored at 420 nm. The enzyme activity was expressed in terms of International Units (1 IU = 1 μmmol ABTS oxidized per min).

### Statistical methods

All statistical analyses were performed using R project software [47]. Principal Component Analysis (PCA) was used to evaluate the correlations among the physicochemical characteristics of the TWs. PCA was conducted using the function *PCA* from the “FactoMineR” package [48].

The temporal trends of microalgae (VSS) and fungi (Colour) cultivations were analysed by means of Multiple Linear Regression (MLR). Time was modelled using a polynomial equation of second order while the impact of diverse characteristics of the TW samples were modelled as their interaction effect with temporal trends of the targeted organisms. The characteristics of the TWs considered in this analysis were: COD, colour, turbidity, concentration of TN-N, NH_4_^+^-N, VSS, metals (Fe, Zn, Ni, Cu, Cr, K, Mg, Ca) and a metalloid (Si). An intercept term representing the value of the dependent variable in the different samples was included in all models to properly represent the initial conditions. The characteristics of the TW samples were considered separately, because the sample size did not allow to fit more complex models while avoiding overfitting. Alternative models (Table 1) were compared by the Akaike Information Criterion, corrected for small sample size (AICc) calculated via the *aictab* function in the “AICcmodavg” package [49]. The difference between the model AICc and the minimum AICc was used to choose the best-fitting model, considering that the model with the lowest AICc generally provides the best description of the data. The characteristics of the textile wastewater samples were deemed to be relevant if the respective model AICc were smaller than the AICc of the model that consider only Time as independent variable and fall within 4 AICc units of the model exhibiting the lowest AICc value [50].

**Table 1:** Formulas used in the multiple linear regression models.

Number	Name	Function
1	Time	*y* = a × *y*_tw_ + poly(Time)
2	Wastewater ID	*y* = a × *y*_tw_ + poly(Time) + poly(Time): Wastewater ID
3	TW *y*	*y* = a × *y*_tw_ + poly(Time) + poly(Time): *x*

Briefly, *x* indicates the characteristics of the TW samples, *y* denotes the response variable, *y*_tw_ is the value of the response variable in the TW samples and Wastewater ID identifies a 7 levels categorical variable representing the different TW samples. All the polynomials used were second order equations; interaction effects are denoted by ‘:’.

The response recovery time, ɳNH_4_^+^-N, ɳTSS and ɳVSS (for microalgae), laccase concentration, biomass dry weight and colour removal (for fungi), spike (for AMX) to the same influent characteristics used for MLR was evaluated using Spearman Rank Correlation. Each metric was separately correlated to each TW sample characteristic and significance (*p-value*<0.05) was calculated for each correlation analysis separately.

During the anammox activity tests, two-ways analyses of variance (ANOVA) were also performed to establish whether the physicochemical characteristics of the TW samples and the number of spikes of nitrite impacted significantly the SAA^max^. Significance level was set at *p-values* lower than 0.05.

## Results and discussion

### Wastewater characterization

The main physicochemical parameters of the wastewater samples are reported and compared with those of the synthetic media used in the microbial assays in Table 2. The values are in agreement with those previously reported for this type of industrial effluents [10]. The highest variability was detected for turbidity and suspended solids. The concentration of total nitrogen and free ammonia varied among samples, while the concentration of ammoniacal nitrogen was quite constant, and the presence of nitrite and nitrate was negligible. As the inorganic nitrogen concentration was constant, the variation of the total nitrogen concentration derived by the organic nitrogen compounds, most probably urea, which is a main reagent of the ink-jet printing process. Likely, the urea demand of the fabric production changed during the sampling campaign causing variations in the concentration of total nitrogen in the wastewater. Shifts of the total nitrogen concentration did affect also the sCOD/N ratio, which ranged between 1.9 and 3.1. High values of such ratio can have severe impact on the anammox reaction, impairing its applicability in the treatment of TW [51]. Fluctuations of the TW composition cannot be avoided, as these are strictly dependent on the manufacturing activity and other types of wastewaters, not linked to the ink-jet printing processes, that are conveyed into the equalization tank. Therefore, such variability should be considered when selecting the treatment process.

**Table 2:** Main characteristic of the synthetic media used in the control tests in the anammox (SM) and microalgae (BBM) assays, and of the seven textile wastewater (TW) samples.

ID	pH	Conductivity (mS cm^-1^)	Turbidity (FAU)	TN-N (mg L^-1^)	NH_4_^+^-N (mg L^-1^)	NH_3_-N (mg L^-1^)	PO_4_^3—^P (mg L^-1^)	COD (mg L^-1^)	sCOD (mg L^-1^)	sCOD/N	TSS (mg L^-1^)	VSS (mg L^-1^)	Colour []
**SM**	7.7	4	NA	330	210	8	5.6	NA	78	4.2	NA	NA	NA
**MBBM**	7.2	2.44	NA	200	200	1.8	53.2	NA	80	2.5	NA	NA	NA
**TW_1**	8.7	1.9	228	190	163	27	1.9	874	590	3.1	220	173	0.29 (purple)
**TW_2**	8.4	3.23	105	181	168	16	2.0	708	472	2.6	118	103	0.174 (green/cyano)
**TW_3**	8.7	2.97	92	291	170	28	2.0	794	556	1.9	115	90	0.245 (green/cyano)
**TW_4**	8.6	2.8	71	243	182	27	1.9	802	587	2.4	100	77	0.232 (green/cyano)
**TW_5**	8.5	2.19	60	145	139	15	1.9	696	538	3.7	97	88	0.139 (green/cyano)
**TW_6**	8.3	2.64	134	169	150	12	1.6	858	532	3.1	115	85	0.182 (green/cyano)
**TW_7**	8.6	2.53	79	156	155	21	2.0	862	454	2.9	90	78	0.237 (green/cyano)
**Mean**	8.5	2.61	110	196	161	21	1.9	799	524	2.8	122	99	NA
**Standard deviation**	0.1	0.45	58	52	14	7	0.1	73	51	0.6	44	34	NA
**CV (%)**	2	17	53	27	9	33	5	9	10	20.5	36	34	NA

NA = not available.

The distribution of metals and metalloids in the wastewater samples is reported and compared with that of the synthetic media in Table 3. Metal contamination in textile wastewater derives from dyes and textile auxiliaries used in the manufacturing chain, depending in their turn on the production requirements. High concentrations of As, Cd, Zn, Cr, Pb, and Hg are hazardous for public health and for the environment [52]. The concentration values of most of the heavy metals and trace elements were quite heterogeneous among samples, with coefficients of variation (CV) mostly over 10%. In the collected TW samples, the concentration values of Pb and Cd were under the detection limit, while those of Cr (0.03 mg L^-1^) and Zn (0.08 mg L^-1^) were within the expected range for this type of wastewater [2,53]. As for colour, similar spectra of absorbance could be observed in all wastewater samples (green/cyano), with the exception of sample 1 (violet/purple).

**Table 3:** Distribution of chemical elements in the synthetic media used in the control tests for anammox (SM) and microalgae (BBM) assays, and in the seven textile wastewater (TW) samples.

ID	K (mg L^-1^)	Mg (mg L^-1^)	Ca (mg L^-1^)	Si (mg L^-1^)	Fe (mg L^-1^)	Na (mg L^-1^)	Zn (mg L^-1^)	Mn (mg L^-1^)	Mo* (mg L^-1^)	Al (mg L^-1^)	Cr (μg L^-1^)	Ni (μg L^-1^)	Pb* (μg L^-1^)	Cu (μg L^-1^)	Cd* (μg L^-1^)
**SM**	7.2	10.2	42.3	ND	2.2	ND	0.12	0.35	0.02	ND	ND	70	ND	80	ND
**MBBM**	88	7.4	5.4	0.23	0.05	78	0.01	0.07	0.03	ND	ND	ND	ND	4	ND
**TW _1**	4.6	8.1	37.9	2.21	0.08	280	0.09	0.012	0.001	0.03	29.0	2.2	0.045	25.0	0.001
**TW _2**	3.5	4.3	18.0	9.04	0.10	228	0.08	0.012	0.001	0.03	43.4	1.7	0.045	66.4	0.001
**TW _3**	4.3	6.9	31.3	3.83	0.16	302	0.11	0.016	0.001	0.03	94.3	2.1	0.045	29.6	0.001
**TW _4**	2.6	6.2	31.2	0.11	0.04	210	0.05	0.007	0.001	0.03	11.4	14.2	0.045	17.9	0.001
**TW _5**	3.9	6.7	31.0	2.15	0.06	247	0.06	0.013	0.001	0.03	24.3	9.2	0.045	34.2	0.001
**TW _6**	4.7	8.1	35.2	1.55	0.08	278	0.07	0.016	0.001	0.03	15.2	7.4	0.045	20.5	0.001
**TW _7**	3.4	6.8	31.5	0.67	0.06	259	0.08	0.011	0.001	0.03	19.7	5.4	0.045	19.3	0.001
**Mean**	3.9	6.7	30.9	2.79	0.08	258	0.08	0.012	NA	0.03	33.9	6.0	NA	30.4	NA
**Standard deviation**	0.7	1.3	6.3	3.00	0.04	32	0.02	0.003	NA.	0.00	28.6	4.6	NA.	16.9	NA.
**CV (%)**	19.2	19.1	20.3	107.48	45.96	12	22.78	24.95	NA	7.35	84.4	76.3	NA	55.7	NA

ND = Value under detection limits.

NA = not available.

**Fig 1:**
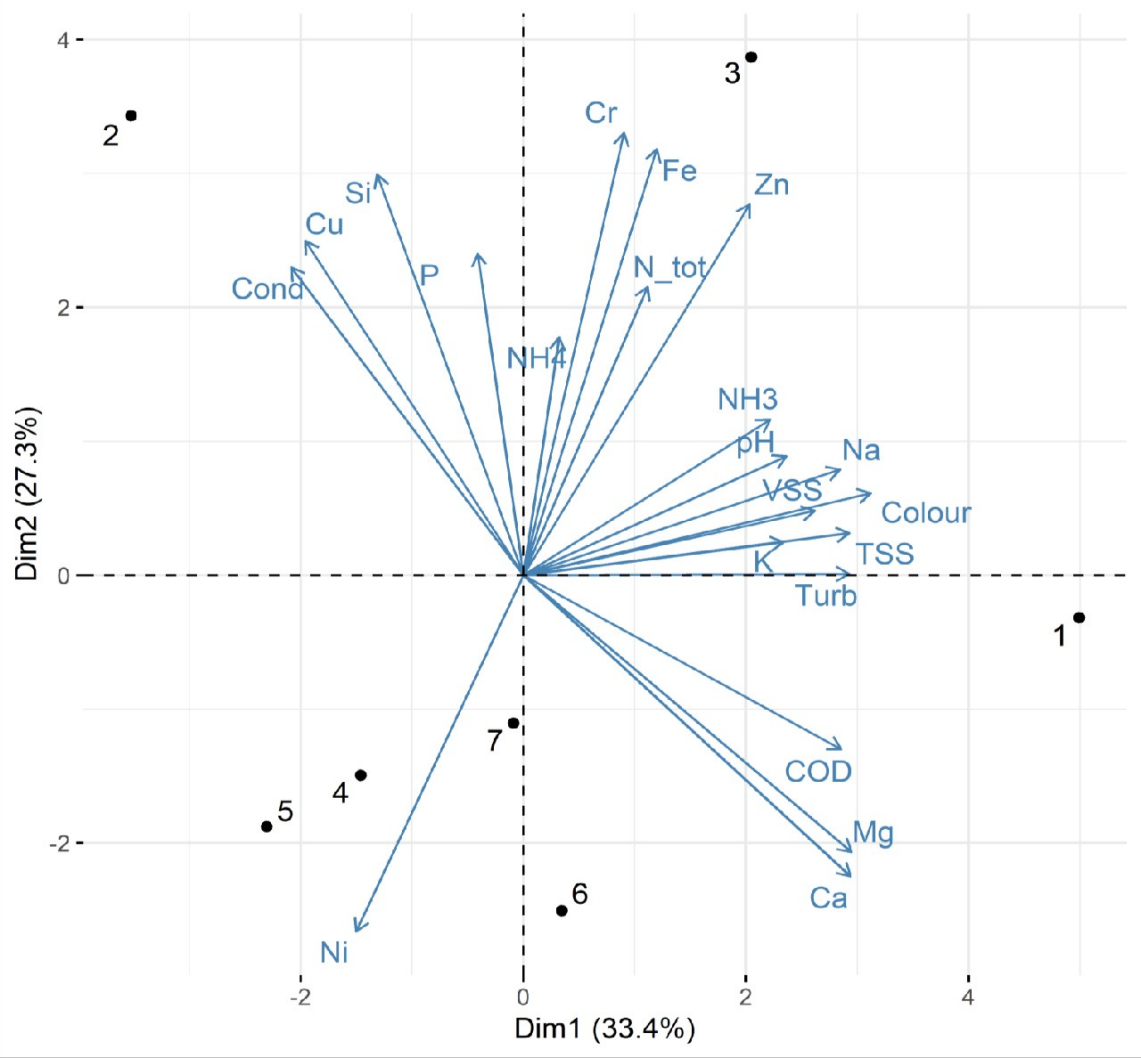
Principal component analysis plot of the physicochemical parameters (arrows) of the seven textile wastewater samples (black dots).

PCA results (Fig 1) demonstrated that the total variance (60.7%) of the physicochemical wastewater characteristics is explained by the first two axis: 33.4% by the first principal component and 27.3% by the second one. The first principal component is positively correlated with the concentrations of Mg, Ca, Na, TSS, as well as with the values of COD, with turbidity and colour. The second principal component correlates positively with the concentration values of Si, Fe, Zn, Cr and PO_4_^3—^P and negatively with the concentrations of Ni. The plots of the two first principal components suggested high orthogonality between the concentration of the major metals, while other elements showed smaller ranges of variation and were not associated with the first two principal components. Such distribution would indicate that the contaminants grouped in the two principal components derive from the same sources within the textile process, but this could be confirmed only by segregating the wastewaters from each process and analysing them, which is out of the scope of the present study.

### Anammox bioassay

**Fig 2:**
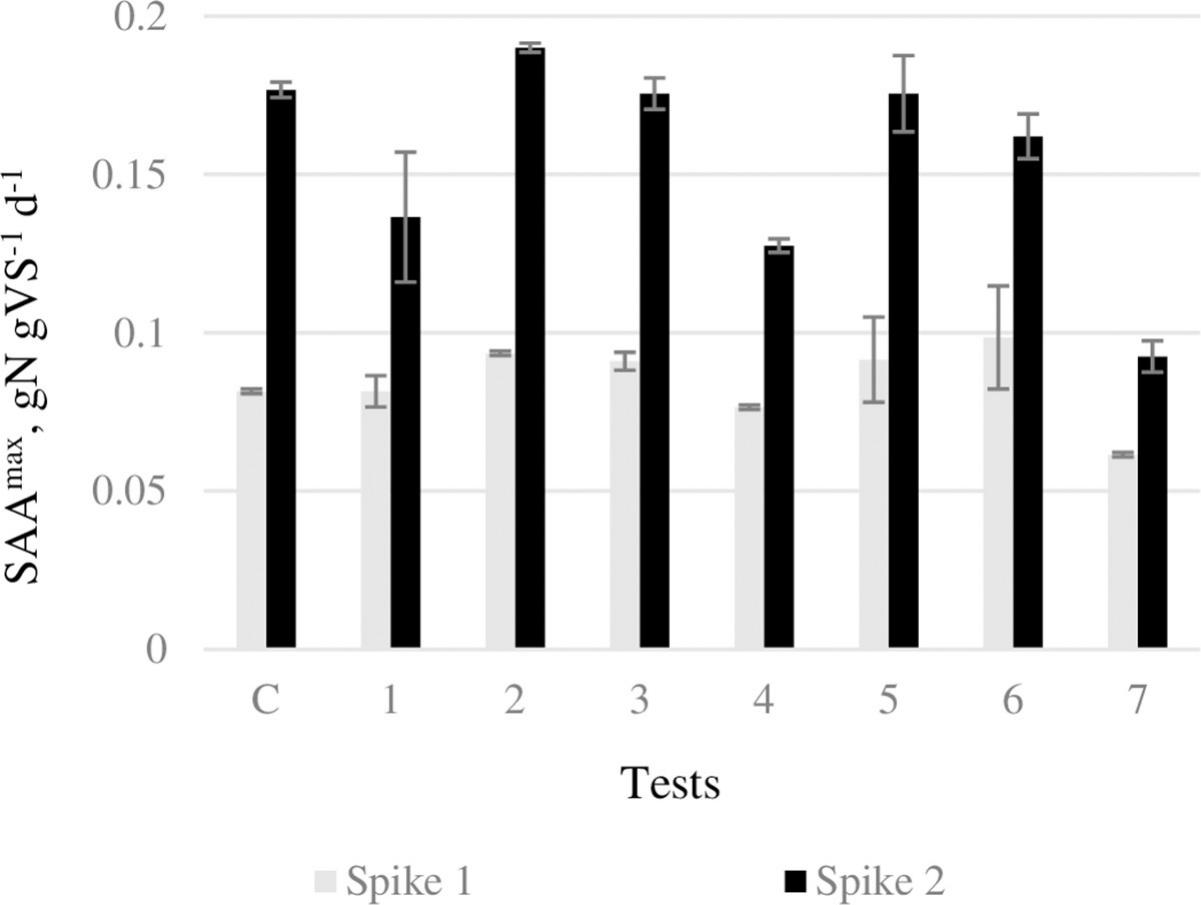
Average maximum specific anammox activities (SAA^max^) achieved after the first (grey bars) and second (black bars) spike of nitrite in the control test (C) and in assays performed with the seven TWs.

Fig 2 summarizes the maximum specific anammox activities (SAA^max^) determined in all short term tests after each injection of nitrite solution. SAA^max^ ranged between 0.06 and 0.09 gN gVS^-1^ d^-1^ after the first spike, but it doubled in most samples after the second one (ranging between 0.09 and 0.19 gN gVS^-1^ d^-1^), except for TW1, TW4 and TW7, where the increase of the activity was more modest. The observed SAA^max^ of the control was low compared to those reported for similar inocula [54]. This suggests that the granular biomass in the inoculum was poorly active possibly due to stressing operational procedures during transportation and storage. The increase in SAA^max^ from the first to the second spike (*p-value*s<0.001; two-way ANOVA) indicates that the test conditions were adequate and favoured the recovery of the anammox bacteria activity. Nonetheless, all SAA^max^ were lower than those reported previously in comparable assays (0.1–0.4 g N g VS^-1^ d^-1^), and in other research focusing on anammox activity in various types of synthetic and industrial wastewaters, such as digestate [10,55].

Considering the chemical composition of the TW samples, the low SAA^max^ activity could not be attributed to the levels of heavy metals and salinity, because all values were well below the IC_50_ reported in the literature [56,57]. In fact, the statistical analyses revealed that the SAA^max^ was positively correlated with the concentration of Si (*ρ* = 083, *p-value* = 0.021) and Cu (*ρ* = 0.92, *p-value* = 0.03). Indeed, metals, at low concentration, are fundamental for the metabolisms of anammox, being components of many enzymes and co-enzymes, such as the nitrite oxidoreductase and nitrite reductase [58].

In addition, the suboptimal sCOD/N ratio (~3) might have contributed to limit the activity of the anammox bacteria by favouring denitrifying bacteria [51] as observed in a long term operation of an PN-anammox sequencing batch reactor, which was fed on textile wastewater taken from the same company [59], or because of the presence of organic inhibitory substances in the oxidizable fraction. Indeed, the statistical analyses revealed that the SAA^max^ was negatively correlated with COD (*ρ* = -076; *p-value* = 0.049). In real applications a preliminary aerobic treatment of the wastewater is required for partial nitritation; this would also decrease sCOD, due to the activity of heterotrophic bacteria, and would probably improve the performances of the downstream anammox process.

In general, the results of this bioassay suggest that some inhibition of the AMX should be expected while processing TW, which may vary depending on the TW characteristics. We should observe that the inhibition response gave only a partial information, as this was referred to short term exposure; tests in a continuous flow system would be more representative of the real biomass activity.

### Microalgal bioassay

Table 4 summarizes the results obtained in the microalgal cultivation tests. The biomass concentration increased significantly only in the control tests reaching 400±39 mg VSS L^-1^ after 15 days, while the VSS concentrations ranged between 113 and 203 mg L^-1^ in the textile wastewater samples. By considering that the VSS of the sole microalgae inoculated in each bottle at T_0_ was 50 mg L^-1^, these findings confirm a strong inhibition of *Chlorella* spp. growth in TW, in agreement with the results of Lim et al. (2010) [19].

**Table 4:** Volatile suspended solids (VSS) in the microalgae cultures and ammoniacal nitrogen removal efficiencies at the end of the tests.

TW	T_15_ VSS (mg L^-1^)	ɳNH_4_-N (%)
1	133	76
2	113	81
3	133	71
4	110	74
5	173	72
6	203	69
7	163	73
C	403	35

The ammonium removal efficiency (ɳNH_4_^+^-N) was higher in the TW tests (74±4%) than in the control ones (35%). However, as the pH in TW cultures was higher than 9 during the experimental trial, stripping may have played a relevant role.

**Fig 3:**
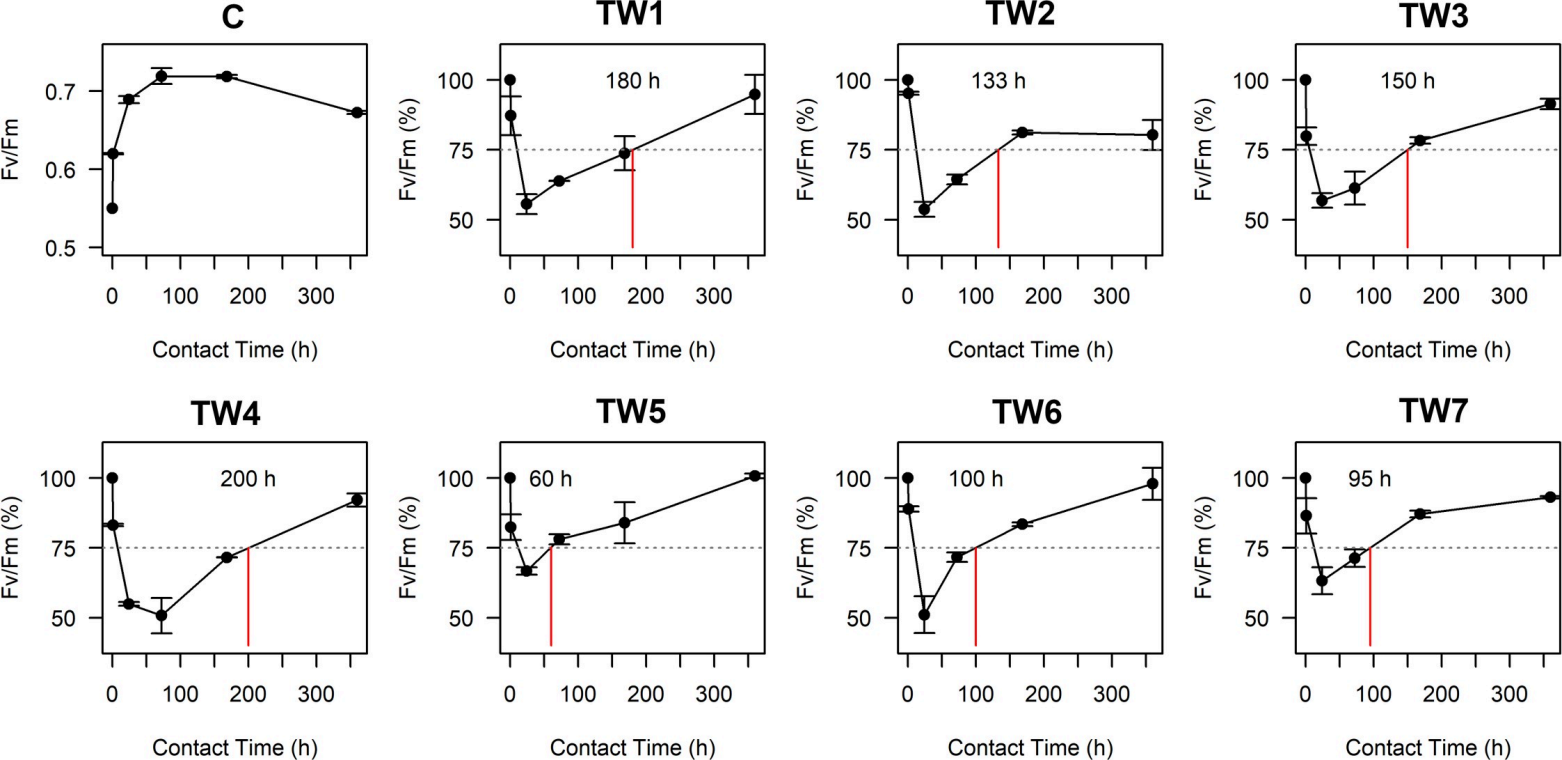
Recovery time representation. Panel C represents the trend of Fv/Fm for the control while the others show the trends of Fv/Fm for each TW, expressed as % of the control. The red lines indicate the recovery time.

**Fig 4:**
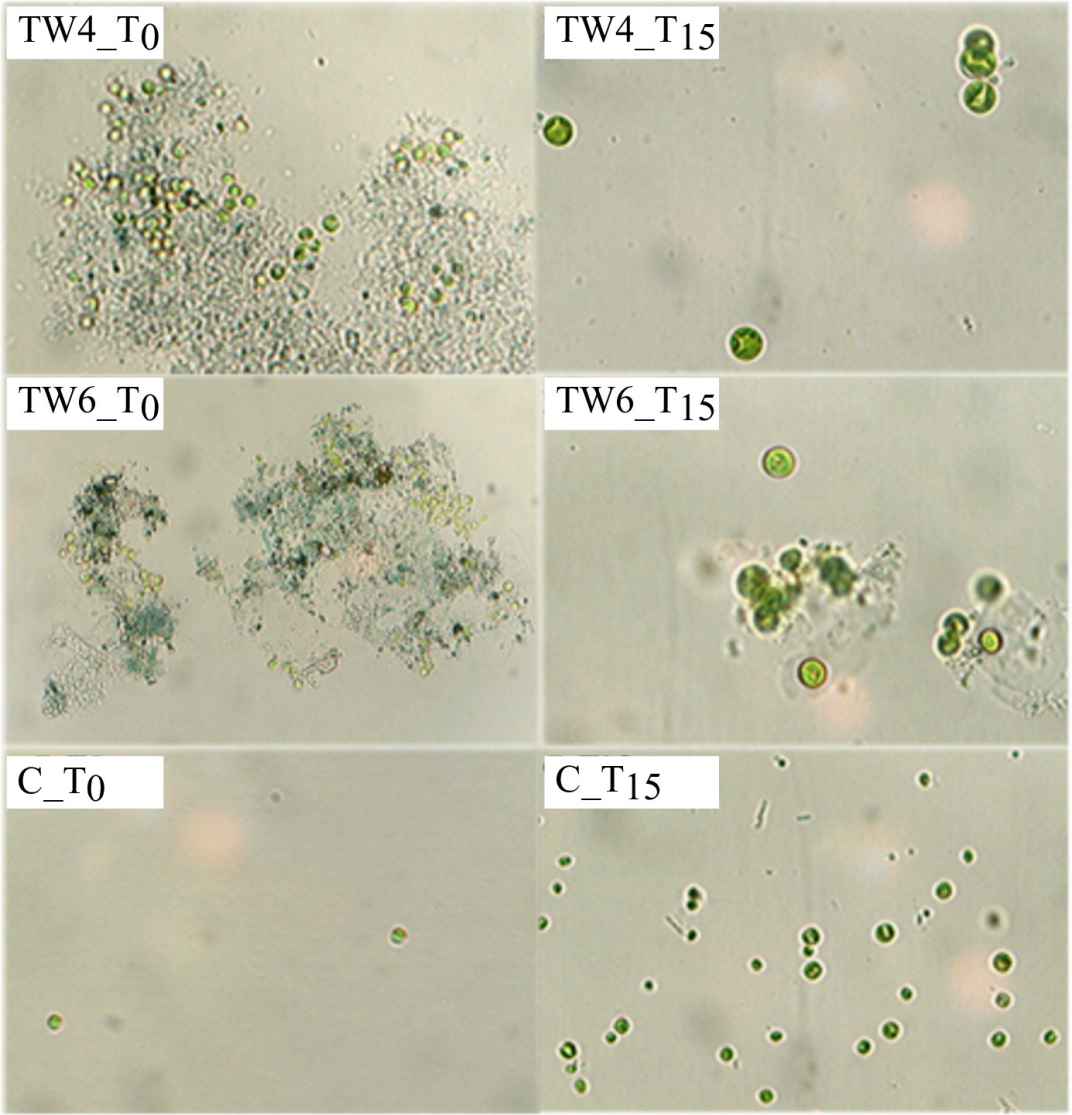
Images of the microalgal cultures in TW4, TW6, and in the control tests (C) captured the day of inoculation (T_0_) and at the end of the tests (T_15_).

Fig 3 shows Fv/Fm values, that estimate the fraction of absorbed quanta used for PS II photochemistry. At the beginning of the tests, Fv/Fm was 0.55; the value of control (C) started to increase after 1 h and remained higher than 0.7 during the first week; a small decrease was observed only at the end of the assay (T_15_: Fv/Fm = 0.675). The high photosynthetic activity in the controls demonstrates that the environmental conditions (i.e. light, mixing and temperature) were adequate for the growth of *C*. *vulgaris*. In the TW tests, Fv/Fm% decreased to ~50% after 24 h of contact time in most samples, indicating a physiological stress, but increased again after 3 days, except for TW5 and TW7, suggesting a gradual acclimatization of the microalgae to the toxic agents in the wastewaters. The recovery times are also reported in Fig 3; the shortest one, meaning the fastest adaptation to the textile wastewater, was observed in TW5 (60 h), while the longest recovery time was measured in TW4 (200 h). The microscopic observation of the microalgal cells during the tests confirmed an immediate shock of the microalgae once transferred in TW samples. Indeed, after few hours from the inoculation most of the biomass was characterized by aggregates of dead and live cells (between 33 and 67% smaller than the controls) surrounded by a coloured matrix (Fig 4). Microalgal self-aggregation is a common response to biotic and abiotic stress conditions, but could also be due to the neutralization of the negative charges on the cell surface by some wastewater component [60]. However, at the end of the trials, singular large (~3.5 time bigger than the controls) microalgae and small definite colonies could be observed suggesting a physiological adaptation of the cells to the TWs. This corroborates previous results showing on the application of a mixed culture of microalgae *(Scenedesmus* and *Chlorella* spp.) for the treatment of TW in a sequencing batch reactor [28]. Nevertheless, further studies should verify whether during the recovering/adaptation time the microalgae develop cellular strategies to face the toxicity of the TW, or whether other microorganisms, such as autochthonous bacteria in the TW, played a role in the biodegradation of part of the recalcitrant and hazardous compounds in the TW facilitating the growth of the microalgae.

In the statistical analyses (Spearman correlation and MLR) the recovery times, ɳ_ABS_, ɳ_VSS_, ɳ_TSS_ and ɳ_N-NH4_ were compared with all the parameters characterizing the 7 TW samples. Spearman correlation revealed that total nitrogen concentration was positively correlated with the recovery time (*ρ* = 0.89, *p-value* = 0.012) and negatively with the ɳTSS (*ρ* = -0.79, *p-value* = 0.036). Likewise, the NH_4_^+^-N concentration was negatively correlated with ɳTSS, ɳVSS and positively with the recovery time (*ρ* = -0.86, *p-value* = 0.014; *ρ* = -0.79, *p-value* = 0.036; *ρ* = -0.86 *p-value* = 0.007, respectively). Solid concentration in the TW seems to have a negative correlation with ɳNH_4_^+^ -N (TSS:*ρ* = -0.79, *p-value* = 0.033 and VSS: *ρ* = -0.81, *p-value* = 0.027). Overall, these findings suggest that nitrogen had a key role in the biomass production and adaptation time. On the other side, the negative correlation between the recovery time and the ɳVSS (*ρ* = -0.86, *p-value* = 0.014, *ρ* = -0.82, *p-value* = 0.023, respectively) confirmed that longer adaptation time causes less biomass production.

**Fig 5:**
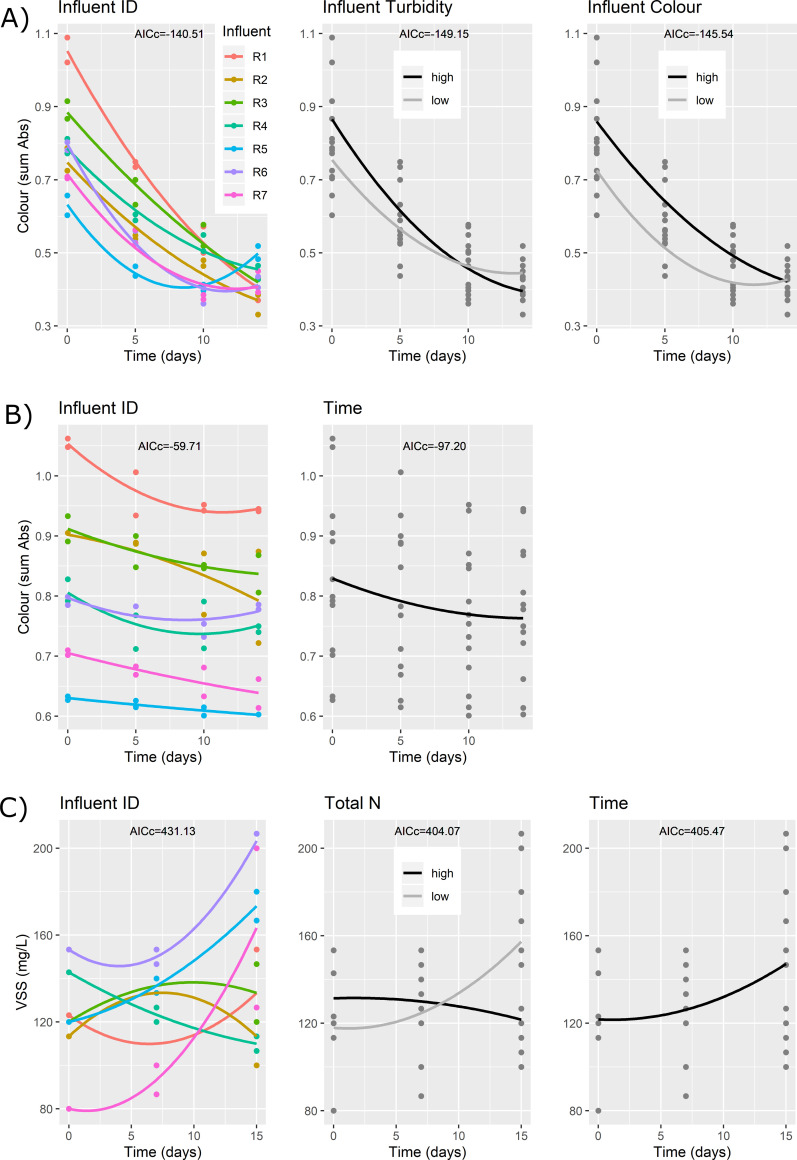
Temporal trend for the fungi (A: *P*. *ostreatus*, B: *P*. *chrysosporium*) and algae (C: *C*. *vulgaris*) cultivations. The first panels on the left represent the average trend for each tested TW sample while the other panels represent the effects of the relevant physicochemical properties identified by the Multiple Linear Regression analyses.

The concentration of VSS in the algal suspensions generally showed an increase, but not in all the tested TW samples (Fig 5C left panel). MLR analyses highlighted a substantial support for the model that considers only Time or the interaction between Time and TN-N (AICc differences smaller than 4). The differences observed among the tests could be due to the concentration of TN-N in the TWs; the VSS increase was larger for lower concentrations of TN-N in the influent and became almost negligible for very high total nitrogen concentrations.

### Fungi bioassays

Tables 5 and 6 report data related to the biomass concentration (g L^-1^) at the end of the tests, culture pH, laccase activity (IU L^-1^) and colour removal efficiency for the each TW of *Pleurotus ostreatus* and *Phanerochaete chrysosporium*, respectively. *P*. *ostreatus* was able to grow in all wastewater samples with a final concentration ranging between 9.10 and 10.12 g L^-1^, without any significant difference among samples. Apart from TW1, the pH at the end of incubation reached similar levels (3.1–3.2), typical of a fungal culture. Laccase activity was observed after 5 and 10 days of incubation and almost disappeared at day 14 (with the exception of TW1). The highest values were observed when the strain was cultivated in TW6 and TW7 (396 and 349 IU L^-1^, respectively). These concentrations were much higher than those reported by An *et al*. [61] in a screening assay examining the laccase production of different strains of *P*. *ostreatus* in submerged (SmF) fermentation. In this study, the maximum laccase activities were evidenced for strain YAASM 0568, with levels ranging from 105.3 to 168.8 IU L^-1^ after 6 to 8 d, in a medium containing 3 g L^-1^ lignin as inducer. Nevertheless, straight comparisons with data in literature are difficult because of discrepancies between fermentation conditions (i.e. submerged -SmF and solid state -SSF fermentation), origin of the effluent to decolorize, and pathways of the enzymatic expression of laccase.

**Table 5:** Summary of the results related to *P*. *ostreatus* ATCC 96997.

TW	Biomass concentration (g L^-1^, 14 d)	Culture pH			Laccase (IU/L)			Colour removal (%)		
		5 d	10 d	14 d	5 d	10 d	14 d	5 d	10 d	14 d
**1**	9.10 ± 0.57	4.9 ± 0.14	4.9 ± 0.11	5.39 ± 0.27	136.0 ± 8.5	32.0 ± 4.2	25.0 ± 4.0	29.6 ± 2.3	49.3 ±2.5	62.0 ± 2.4
**2**	10.07 ± 0.38	7.25 ± 0.21	4.49 ± 0.16	3.15 ± 0.21	126.7 ± 5.0	23.8 ± 2.3	<0.5	28.0 ± 5.1	37.4 ± 5.1	52.7 ± 2.3
**3**	9.79 ± 0.30	7.11 ± 0.08	4.79 ± 0.14	3.14 ± 0.08	195.0 ± 7.1	193.3 ± 2.4	<0.5	25.1 ± 8.2	38.5 ± 2.3	54.0 ± 1.1
**4**	10.12 ± 0.18	7.22 ± 0.11	4.10 ± 0.14	3.24 ± 0.17	87.5 ± 3.5	89.4 ± 3.9	<0.5	24.5 ± 4.1	33.3 ± 6.3	43.6 ± 5.4
**5**	9.56 ± 0.20	7.17 ± 0.08	3.52 ± 0.17	3.14 ± 0.06	190.0 ± 7.1	184.7 ± 6.0	<0.5	28.5 ± 1.4	35.5 ± 5.7	20.2 ± 8.9
**6**	9.80 ± 0.28	7.16 ± 0.06	3.5 ± 0.07	3.25 ± 0.21	395.6 ± 12.1	376.4 ± 5.1	<0.5	31.3 ± 1.5	51.7 ± 3.0	46.9 ± 3.7
**7**	9.43 ± 0.17	7.26 ± 0.11	3.34 ± 0.06	3.24 ± 0.13	349.4 ± 16.2	333.9 ± 8.6	<0.5	23.0 ± 3.5	46.3 ± 1.0	40.4 ± 6.0

**Table 6:** Summary of the results related to *P*. *chrysosporium* DSM 9620.

TW	Biomass concentration (g L^-1^, 14 d)	Culture pH			Laccase (IU L^-1^)			Colour removal (%)		
		5 d	10 d	14 d	5 d	10 d	14 d	5 d	10 d	14 d
**1**	10.48 ± 0.68	4.21 ± 0.13	4.55 ± 0.10	5.07 ± 0.18	<0.5	<0.5	<0.5	8.1 ± 4.0	10.2 ± 1.5	10.6 ± 0.6
**2**	8.45 ± 0.78	4.31 ± 0.04	3.97 ± 0.04	2.76 ± 0.13	<0.5	<0.5	<0.5	1.1 ± 0.9	8.7 ± 5.0	11.2 ± 1.0
**3**	8.14 ± 0.51	6.02 ± 0.08	2.85 ± 0.11	2.94 ± 0.06	<0.5	<0.5	<0.5	4.2 ± 0.9	6.9 ± 2.6	8.3 ± 1.8
**4**	7.80 ± 0.57	4.34 ± 0.08	3.92 ± 0.06	2.71 ± 0.13	<0.5	<0.5	<0.5	8.7 ± 2.1	7.2 ± 3.9	8.0 ± 2.0
**5**	8.13 ± 0.38	4.33 ± 0.08	4.06 ± 0.08	2.89 ± 0.08	<0.5	<0.5	0.5	1.5 ± 1.9	3.5 ± 0.9	4.3 ± 0.6
**6**	7.86 ± 0.62	4.32 ± 0.35	3.40 ± 0.42	3.09 ± 0.01	<0.5	<0.5	<0.5	1.1 ± 1.2	6.2 ± 0.8	1.3 ± 0.5
**7**	8.41 ± 0.62	4.42 ± 0.30	3.12 ± 0.10	3.02 ± 0.18	<0.5	<0.5	0.7	4.3 ± 0.6	7.0 ± 4.1	9.7 ± 4.1

Colour removal ranged from 20% (TW5) to a very interesting 62% (TW1); in all the other tests decolorization was around 40–54%. The moderate and varied colour removal suggests that the tested *Pleurotus* may have been sensitive to undetected compounds present in some samples, i.e. trace metals and textile auxiliaries that can stimulate and/or inhibit enzymatic activities. This might also explain the divergences between the colour removal efficiency observed when using real TW or solution with synthetic dyes [62].

MLR (Fig 5) analyses highlighted that turbidity and initial colour interacted with Time for the best fitting models. Higher values of turbidity, as well as colour, were associated with higher colour removal. Laccase, colour removal and biomass growth were all compared with the parameters characterizing the 7 TWs. Results showed that colour removal was positively correlated with the presence of Iron in the textile wastewater samples (*ρ* = 0.77, *p-value* = 0.041). Iron, together with Copper, have been already reported to induce the enzymatic activities correlated to colour removal of cherry waste by in *Pleurotus eringii* [63].

Different performances emerged in tests inoculated with *P*. *chrysosporium*. This strain was able to grow in all effluents with final biomass concentration ranging between 7.8–10.5 g L^-1^. A consequent pH decrease could be observed in the cultures. Nevertheless, laccase enzymatic activity was never detected and a limited colour removal (10–11%) was evidenced. Kiran et al. [64] investigated the colour removal performance of four *P*. *chrysosporium* strains on a dye wastewater solution prepared by mixing Reactive Blue, Reactive Violet and Reactive Yellow: after 6 day of incubation, the decolorization efficiency ranged from 38.9 to 82.9% depending on the tested strain and on the concentration of synthetic dyes, suggesting that larger quantity of the dye may cause a slower rate of dye removal; contrarily to results obtained in the present experimentation, laccase was always found in the range 5–25 IU mL^-1^. This comparison suggests that *P*. *chrysosporium* was able to grow but not to synthetize the enzymatic activity requested for colour removal.

Laccase production by *P*. *chrysosporium* is reported to be highly related to the conditions of cultivation of the fungus, while high biomass did not necessarily lead to high laccase yields [65]. Sedighi et al. [66] highlighted that maximum decolorization of Astrazon Red FBL (87%) and COD removal (42%) of a textile effluent by *P*. *chrysosporium* occurred only when Tween 80 (0.05%, w/v) was added to the effluent. Other authors advocate the possibility of raising laccase production by increasing nitrogen concentration or when carbon or sulphur become limiting [65].

MLR analyses confirmed that the decolorization trends were similar among the trials and the final values depended only on the initial colour values and Time. Pairwise comparisons between colour removal, biomass growth and the parameters of the tested TW samples showed that colour removal was positively correlated with VSS (*ρ* = 0.76, *p-value* = 0.049), while biomass growth was negatively correlated with Nickel concentration (*ρ* = 0.79, *p-value* = 0.048). The first result can be easily explained by the fact that VSS is directly linked with colour removal; the more fungus is present, the higher the removal achieved; as regards the second result, even if Ni is an essential metal, at micromolar concentration it can inhibit the growth of *P*. *chrysosporium* [67].

In summary, these results suggest that significant decolorization was observed in all samples with variable performance depending on the TW samples and fungal strain. However, *P*. *ostreatus* resulted more promising than *P*. *chrysosporium* in TW decolorization.

## Conclusions

The wastewaters originated by DTP technology showed a significant variability in characteristics. Nonetheless, none of the tested samples showed extreme toxicity effects, which would hinder the applicability of the studied bioprocesses. Smoother responses to variable wastewater characteristics are to be expected in scale up tests and the continuous operation mode should promote bioadaptation and improve the buffering capacity of the system through the bioreactors retention time.

The treatment performance could be improved by integrating the tested microbes in a treatment train where fungi would reduce COD and colour, a PN/anammox phase would reduce the ammoniacal nitrogen, and a final microalgae-based photobioreactor would act as a finishing step for further nitrogen reduction and to produce biomass with added-value (i.e. pigment content). The produced pigments could potentially be reused as green ingredients in the printing process, thus implementing the circular economy principles.

## Supporting information

S1 Fig OxiTopControl bottles used for the anammox activity tests. (TIF)
