# Supplementary figures and images for: Assessment of anammox, microalgae and white-rot fungi-based processes for the treatment of textile wastewater
Source: PLoS One. 2021 Mar 2;16(3):e0247452. doi: 10.1371/journal.pone.0247452 (PMC7924738; doi:10.1371/journal.pone.0247452)

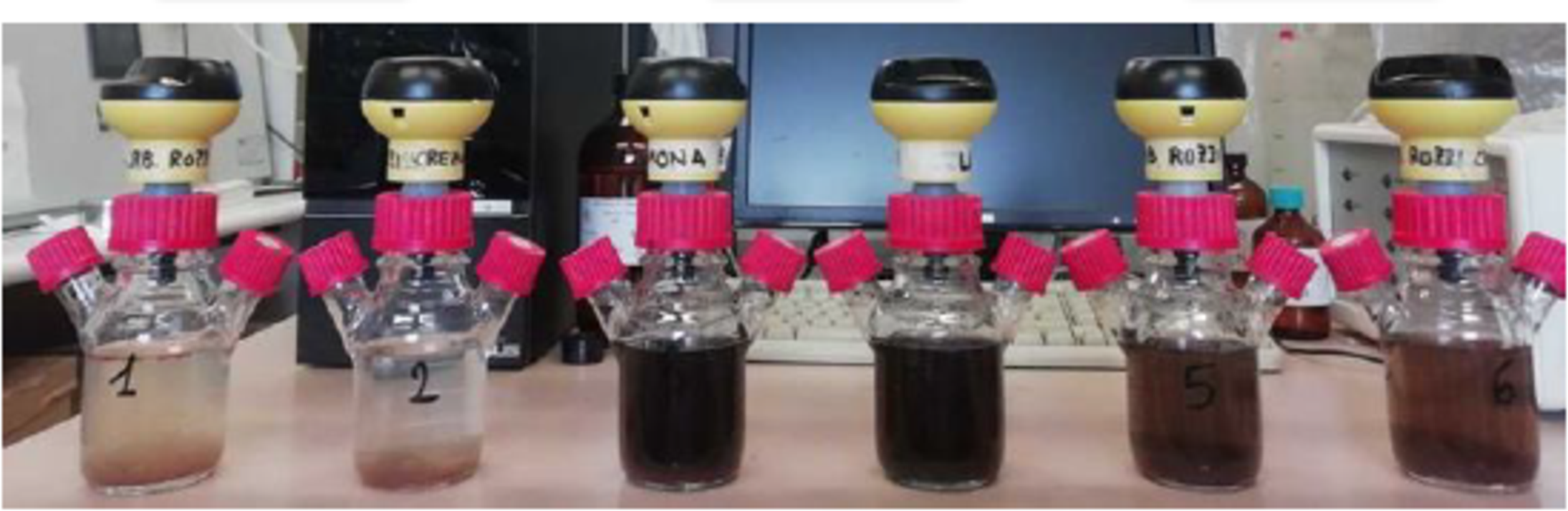

Supplement: S1 Fig — (TIF) [file pone.0247452.s001.tif]
